# Genome bioinformatic analysis of nonsynonymous SNPs

**DOI:** 10.1186/1471-2105-8-301

**Published:** 2007-08-20

**Authors:** David F Burke, Catherine L Worth, Eva-Maria Priego, Tammy Cheng, Luc J Smink, John A Todd, Tom L Blundell

**Affiliations:** 1 Department of Biochemistry, University of Cambridge, 80 Tennis Court Road, Cambridge, CB2 1GA, UK; 2 Juvenile Diabetes Research Foundation/Wellcome Trust Diabetes and Inflammation Laboratory, Cambridge Institute for Medical Research, University of Cambridge, Cambridge, CB2 2XY, UK

## Abstract

**Background:**

Genome-wide association studies of common diseases for common, low penetrance causal variants are underway. A proportion of these will alter protein sequences, the most common of which is the non-synonymous single nucleotide polymorphism (nsSNP). It would be an advantage if the functional effects of an nsSNP on protein structure and function could be predicted, both for the final identification process of a causal variant in a disease-associated chromosome region, and in further functional analyses of the nsSNP and its disease-associated protein.

**Results:**

In the present report we have compared and contrasted structure- and sequence-based methods of prediction to over 5500 genes carrying nearly 24,000 nsSNPs, by employing an automatic comparative modelling procedure to build models for the genes. The nsSNP information came from two sources, the OMIM database which are rare (minor allele frequency, MAF, < 0.01) and are known to cause penetrant, monogenic diseases. Secondly, nsSNP information came from dbSNP125, for which the vast majority of nsSNPs, mostly MAF > 0.05, have no known link to a disease. For over 40% of the nsSNPs, structure-based methods predicted which of these sequence changes are likely to either disrupt the structure of the protein or interfere with the function or interactions of the protein. For the remaining 60%, we generated sequence-based predictions.

**Conclusion:**

We show that, in general, the prediction tools are able distinguish disease causing mutations from those mutations which are thought to have a neutral affect. We give examples of mutations in genes that are predicted to be deleterious and may have a role in disease. Contrary to previous reports, we also show that rare mutations are consistently predicted to be deleterious as often as commonly occurring nsSNPs.

## Background

The recent sequencing of the human genome has provided a wealth of information detailing several million genetic variations between individuals. This offers new opportunities for identifying the genetic predisposition to and understanding the causes of common diseases. It has been estimated that 90% of genetic variations in humans are due to single nucleotide polymorphisms (SNPs) [1], most of which have minor allele frequencies exceeding 0.05 and will provide a significant proportion of common causal variants that will be mapped and identified in the future. Through the HapMap project over 4 million of these have been genotyped in a common panel of DNA samples, not only validating the SNP and estimating its allele frequency in the general population, but also assessing the degree of linkage disequilibrium (LD) between them [2,3]. Moreover, SNP genotyping technologies have advanced recently to the point that hundreds of thousands of SNPs can be typed in thousands of individuals, for example using the case-control design [4]. Hence, the discovery of causal variants for common diseases is set to accelerate and it would be advantageous if the functional effects of SNPs could be predicted bioinformatically, in order to direct functional studies and narrow down the best candidate SNPs in regions of the genome that show high LD.

The most identifiable category of SNP is the small proportion (less than 1%) that change protein sequence, the most common of which is the non-synonymous SNP. There are now several databases that catalogue these variations, such as the human genome variation database, HGVBase, [5,6] and the National Center for Biotechnology Information (NCBI) database of SNPs, dbSNP [7,8]. Most of these sequence variations have been identified by sequencing and genotyping DNA samples from general populations rather than disease groups, most particularly in the HapMap project [2], and using the new genotyping technologies panels of genome-wide nsSNPs are now being studied in disease and control populations [4,9,10]. The Human Gene Mutation Database (HGMD) [11,12] collates known gene mutations responsible for human inherited disease. Similarly, the genetic association database (GAD) [13] has archived over 3,600 dbSNP and HGVBase entries that have reported disease associations from published clinical studies, although very few of these statistical associations are validated. The Mendelian Inheritance in Man (MIM) database is a catalogue of genetic disorders of inherited diseases mapped to human genes and highly penetrant, but rare (MAF < 0.01) mutations [14,15].

Knowledge of the three dimensional structure of a gene product is of major assistance in predicting and understanding its function, its role within the cell and its role in disease. Over the past few years, there have been many computational methods developed to predict whether a mutation is deleterious to the structure or the function of the gene and will therefore lead to disease. In general, these classify the mutations into whether they have negative, neutral or positive effects on the structure or function of the proteins. There are several methods that try to estimate loss (or gain) in energy of the protein structure due to single sequence changes. The method developed by Topham and colleagues, Site Directed Mutator (SDM), is based upon environment substitutions using an analogy to the thermodynamic cycle [16]. The Mupro method [17] uses support vector machine learning to predict protein stability changes for single amino acid mutations using both sequence and structural information, as does the I-Mutant2.0 method [18,19].

Several methods use sequence conservation of particular amino acids within a family of sequences or look for particular features of a protein structure to predict whether a substitution affects protein function. For example, SIFT (Sorts Intolerant From Tolerant substitutions) [20-22] distinguishes those residues that are conserved amongst homologous sequences, and, thus, intolerant to most sequence changes, from those residues that are poorly conserved and tolerant to sequence changes. SIFT does not require structural information and, therefore, can be applied to protein sequences in general.

Another resource, the LS-SNP database [23,24], contains predictions based on machine learning techniques, of whether a nsSNP will have an effect on protein-ligand binding or have a deleterious impact the function of the protein and would thus lead to disease. The current release of LS-SNP (2005-09-02) predicts that, out of nearly 21,000 nsSNPs extracted from dbSNP, over 4,700 (22%) of the nsSNPs will destabilize protein structure or interfere with either ligand binding or the formation of domain-domain interfaces [23]. Predictions of the POLYPHEN method are based on empirical rules based on the sequence, phylogenetic and structural information characterizing the substitution [25-27]. Ramensky *et al. *predicted that 25% (2,848 out of 11,152) of the nsSNPs from HGVBase (version 12) would be damaging [26].

Functional information, detailing which residues are involved in catalysis, binding of ligands or interactions with other proteins, is sometimes available in databases or in the literature. The Catalytic Site Atlas [28] details residues involved in enzyme catalysis for nearly 15,000 proteins based on over 700 literature reports. Many computational methods for the prediction of which residues may be involved in function have also been developed [29-32]. A widely used computational technique to predict functional residues, based upon the evolutionary conservation of sequences, is the "evolutionary trace" method [33-35]. In this technique, phylogenetic information based on homologous sequences is used to rank residues by evolutionary importance which are conserved in a protein sequence. This conservation can then be mapped onto a representative structure. Clusters of conserved residues in three dimensions can detect functional surfaces such as those involved in protein-protein interactions. The 3DCA method [29] extended this to identify spatial clusters of residues that are more conserved in sequence than expected given their overall sequence identity. However, the conservation of amino acid residues has been shown to be strongly dependent on the environment in which they occur in the protein structure [36,37]. Methods that use conservation of sequence do not exclusively distinguish between evolutionary restraints arising from the need to conserve function, from those that arise from the conservation of the structural environment. Since the core of the protein is likely to be conserved for structural reasons, these methods often only consider residues on the surface of the protein. This approach is particularly problematic for catalytic residues of enzymes, which can often be relatively inaccessible to solvent.

The Crescendo method identifies those residues that have a higher degree of conservation than would be expected on the basis of the local structural environment [30]. It is assumed that these additional restraints on allowed amino acid substitutions are due to particular functions mediated by interactions with other molecules. Once potential functional residues have been identified, known mutations can be mapped onto the structure of the protein, if known, and a prediction can be made of their effect on function. For instance, if a residue is a known catalytic residue or is close to a known binding site, mutations of this residue are likely to affect function.

In the present report we detail the large-scale automated homology modelling of 6,000 genes that were selected by us as functional candidate genes in immune-mediated disease and the subsequent analysis of 24,000 nsSNPs found within these genes. The goal of the analysis is to predict a subset of mutations which are likely to affect the structure or function of the gene, and thus to identify which of these mutations may have a role in the progression or development of inflammatory or immune diseases.

## Results

### Genome-wide prediction of protein structure

Table 1 shows a summary of available databases detailing sequence variation in the human genome. Two datasets of nsSNPs were selected and analysed, 2,249 sequence changes found in 500 genes in OMIM and 21,471 nsSNPs in 5,500 genes from dbSNP125. It is well acknowledged that buried residues tend to be more conserved in evolution and mutations of these residues would be expected have a marked effect of the structure of the protein, resulting in an increased risk of causing disease. Nevertheless, residues on the surface of the protein are more likely to be involved in protein-protein or protein-ligand interactions. Unfortunately, structural information is usually restricted to a small proportion of the genes for which experimental crystal structures have being determined. To increase the coverage of structural information for these genes, we implemented an automated, large-scale, structure prediction strategy using homology modelling. This technique requires a prior prediction of the likely fold of the protein, often termed homology or fold recognition.

**Table 1:** Databases used. Summary of databases mentioned in this analysis

Database	Description	nsSNPs used for this study	Availability
dbSNP	Database of Single Nucleotide Polymorphisms	21471	[8]
OMIM	Online database of Mendelian Inherited dimorphisms in Man	2256	[15]
HapMap	Database quantifying frequencies of common haplotypes in four populations	5770	[3]
HGMD	Human Gene Mutation Database of mutations within the coding regions, splicing and regulatory regions of human genes causing inherited disease	N/A	[12]
GAD	Genetic association database of medically relevant polymorphisms identified in published scientific papers	N/A	[13]
HGVBase	Human Genome Variation Database	N/A	[6]
LS-SNP	Database of large scale annotation of predicted effects of human SNPs.	11220	[24]
Polyphen	Database of predicted functional effect of human nsSNPs	4459	[27]
HOMSTRAD	Database of protein structures classified by protein family	N/A	[40]

**Figure 1:**
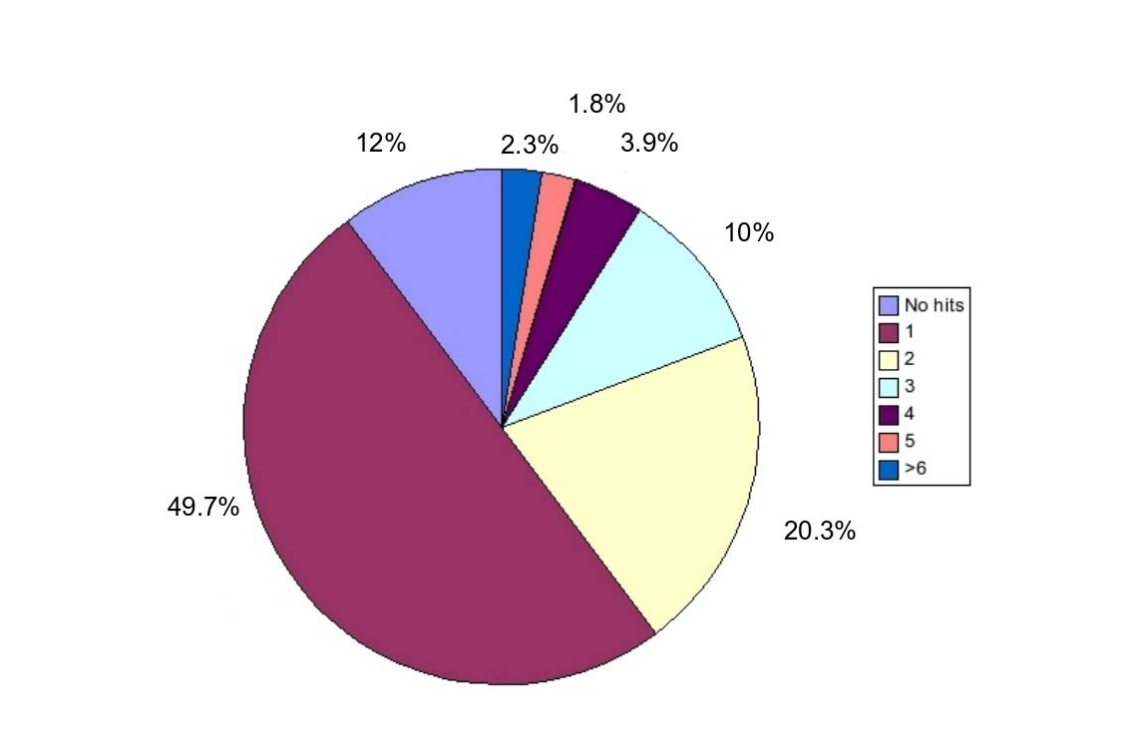
**Distribution of fold recognition results**. Distribution of the number of HOMSTRAD families predicted by FUGUE for a gene.

Automated homology recognition for all of the genes was performed using FUGUE [38,39]. The average length of the protein sequence within the list of genes is 400 residues with more than 95% of the sequences having fewer than 1,000 amino acids. A few genes are exceptionally long, such as the SYNE1 gene (nuclear envelope spectrin repeat protein), which contains over 8,800 amino acids. Figure 1 shows the distribution of the number of HOMSTRAD [40] families per gene that were predicted by FUGUE. Surprisingly, only 661 (12%) of the genes had no significant FUGUE prediction (Z-score < 7). There was a single HOMSTRAD family predicted for 2,727 (~50%) of the genes. This does not imply a single structural domain within the genes since some of the HOMSTRAD families represent multiple structural domains. Two HOMSTRAD families, and thus multiple domains, were predicted in 1,117 (~20%) of the genes and three families for 551 (~10%) of the genes. More than 99% of the sequence alignments produced by FUGUE have a length of less than 500 residues.

### Homology recognition of protein sequences

Over 16,000 models were built for over 4,700 genes that had significant FUGUE scores, using the program Modeller[41-43]. For 37% of the genes, there were multiple FUGUE scores representing multiple structural domains in the gene. In these cases models representing each structural domain were built with no attempt to join these into a single model for the gene. The percentage of sequence variations for which a structure can be predicted varied between the two datasets. Over 62% of mutations from the OMIM (1,403 nsSNPs in 375 genes) were in a region of the gene with a structure prediction in contrast to only 51% of the dbSNP (11009 nsSNPs in 3,956 genes).

The relative solvent accessible area (ASA) was calculated for all residues in the models. Over 58% of the amino acids with structures that have OMIM mutations are buried (<40% ASA), compared to only 40% of the mutations in dbSNP. These mutations would, therefore, be expected to affect the structure or folding of the proteins. These values are in good agreement with a smaller study of 63 crystal structures of human proteins with disease causing mutations [44]. It would also suggest that 40% of the OMIM mutations are exposed to solvent and may disrupt protein interactions rather than disrupt the structure or folding of the protein. On the contrary, most of the mutations in dbSNP, which are expected to have a neutral effect on the structure and function of the protein, tend to occur on the surface of the protein where amino acid substitutions are more readily accommodated.

### Prediction of deleterious mutations

Although several methods are available for prediction of functionality, they are mostly not readily usable for the large number of nsSNPs in genome-wide analysis. Generally, only a small number of the published predictions correspond to the mutations found in our datasets. We have, therefore, focused only on methods for which we could either download pre-computed predictions for a large proportion of our datasets or where we were able to implement the methods in-house. These methods and the total number of nsSNPs analysed for each method is summarised in table 2.

**Table 2:** Prediction methods. Summary of computational methods mentioned in this analysis

Method	Predicts effect on Structure	Predicts effect on Function	Requires structural information	Method	Number of nSNPs with predictions	Availability
SIFT	Implicit	implicit	No	SequenceConservation	22728	[22]
LS-SNP	Yes	yes	No	Knowledge-based rules and support vector machine	11220	[24]
POLYPHEN	yes	yes	Yes	Sequence Profile and structural properties	4459	[27]
SDM	Yes	no	Yes	Structure based Substitution Table	5705	Available On request
Imutant	yes	no	Yes	Support vector machine	8879	[19]
env_score	Yes	no	Yes	Structure based Substitution Table	10625	Available On request
Crescendo	No	yes	Yes	Structure based Substitution Table	10625	Available On request
FUGUE	N/A	N/A	No	Sequence Substitution Profile	N/A	[39]
Modeller	N/A	N/A	Yes		N/A	[43]

Cross-referencing the pre-computed LS-SNP predictions, we find LS-SNP predictions for 52% (11220 nsSNPs in 2381 genes) of our dbSNP dataset. In total 10% (2,291 nsSNPs in 1,511 genes) are predicted to affect either the structure or function of the gene. Polyphen predictions were only available for 21% of dbSNP (4,459 nsSNPs in 2,237 genes). Just under 25% of these predictions (1,141 nsSNPs in 845 genes), or 5% of the total, were predicted to be damaging. Less than 3% of the SNPs (549 nsSNPs in 367 genes) have both LS-SNP and Polyphen predictions. Both methods agree on a deleterious prediction for only 1% of the dbSNP (225 nsSNPs in 203 genes). There was no data available for the sequence variations in the OMIM for either LS-SNP or Polyphen.

When Ng and Henikoff applied SIFT to a set of nsSNPs annotated in SWISS-PROT, 69% (3,626/5,218) were correctly predicted as damaging [45]. SIFT also predicted that 25% of the sequence variations in dbSNP will affect protein function. However, they estimated the false positive rate to be 20%, suggesting that most nsSNPs in dbSNP are functionally neutral. We applied the SIFT algorithm to our datasets using the sequence alignments generated from the FUGUE fold recognition algorithm. Nearly 51% of the nsSNPs in OMIM (1,138 nsSNPs in 330 genes) were predicted to be deleterious. This is lower than Ng and Henikoffs original estimate for disease related mutations. This difference is in part due to the use of FUGUE derived sequence alignments, which generally produces more accurate sequence alignments than most methods. Additionally, the number and divergence (and hence sequence conservation) of homologous sequences has increased since their original study. Both of these factors will help to reduce false positive predictions. In agreement with their original study, we find that 25% of nsSNPs from dbSNP(5,506 nsSNPs in 2,766 genes) are predicted to be deleterious. Comparing these predictions with other methods may prove useful in further removing false positive predictions. Almost one sixth, or 4% of mutations in dbSNP (851 nsSNPs in 651 genes), are predicted to be deleterious by both SIFT and LS-SNP. SIFT and Polyphen predictions, however, only agree for 2% (495 nsSNPs in 391 genes) of dbSNP. Considering all three methods, the number of nsSNPs that are predicted to be deleterious drops to 0.6% (124 in 114 genes) of dbSNP. This figure represents about 3% of the total number of the nsSNPs that have Polyphen predictions, largely in line with the 5% true positive estimate given by Ng and Henikoff. If Polyphen predictions were available for the whole dataset, we should, therefore, expect around 1,000 nsSNPs to be predicted as deleterious by all three methods.

### Environment substitution scores

Several methods have been developed to estimate the probability of finding a given amino acid substitution in a given structural environment [36,37,46,47]. Commonly used definitions of the structural environments include the accessibility of the side-chain of the amino acid to solvent; the conformation of the backbone of the amino acid (helix, strand, coil); and whether the amino acid form hydrogen bonds to other amino acids or ligands. Tables of the log-odds probability of finding a given amino acid substitution, in a given structural environment, have previously been calculated for a variety of structural environments [36,37]. These tables describe the likelihood of a sequence mutation being acceptable within a given environment, and thus observed in homologous sequences. Given a model of the protein structure, we can calculate the environmental score (env_score) for each mutation in the datasets. A negative score would suggest that the given mutation is not accommodated without major structural and environment changes to the protein and can be defined as deleterious. A score close to zero is neutral to the mutation whereas a positive score suggests that the substitution is not only readily accepted within the environment but favoured in evolution.

Since there is not structural information available for all regions of the genes, there are env_score for only 45% of nsSNPs in dbSNP (9962 in 3198 genes) and 60%(1355 in 355 genes) of OMIM mutations. Over 16% of the OMIM mutations have very high negative scores (env_score < -4) suggesting a major, and, thus, deleterious structural change is required to accommodate these mutations. In total, nearly one third of the OMIM mutations (35%) are negative (env_score < -1) with 50% predicted to be favourable (env_score > 1). In contrast, only 6.6% of the mutations in the dbSNP have extremely negative scores; 18% negative and 69% positive scores. Env_score and Polyphen both predict substitutions to be deleterious for 1.7% (372 in 308 genes) of the dbSNP dataset. LS-SNP and env_score show a slightly higher agreement of 3.6% (781 in 592 genes). All three methods agree for only 106 nsSNPs.

The most common of these unfavourable substitutions are mutations from a cysteine, usually buried, to a large hydrophobic residue (often tryptophan or phenylalanine). Also common is a proline to leucine substitution as well as mutations from a glycine residue. For example, the nsSNP, Thr455Ile (rs870849; MAF_ceu _= 0.408%), in the lymphocyte activation gene-3 (LAG3) gene is predicted to be deleterious by env_score, LS-SNP and Polyphen. This gene is involved in lymphocyte activation and is known to bind to HLA class II antigens, and the nsSNP has been reported to be associated with susceptibility to multiple sclerosis [48].

### Prediction of structural stability

In this study, we have used two methods (SDM[16] and Imutant[18]) that quantitatively estimate the effect of a mutation on the stability of a protein structure. Both methods require a model of the structure of the protein and are thus rarely used in such genome wide studies of nsSNPs. SDM predicts nearly twice as many (11%) of the disease causing mutations in OMIM to have ΔΔG scores less than -3 than those mutations in dbSNP (6%). The mutation with the lowest predicted SDM energy score (-12.4) is the R149C sequence substitution in the OMIM found in the Xanthine dehydrogenase (XDH) gene. This is also predicted to be deleterious by SIFT, env_score (-9) and slightly damaging by Imutant(-1.05). This mutation is linked to the condition of type I Xanthinuria, which is caused by XDH deficiency. Another mutation in this gene (P1150R) is also predicted to be structurally deleterious by env_score(-3) and SDM (-5.9).

An equally damaging and identical amino acid substitution (R573C), also found in OMIM, occurs in the adenosine monophosphate deaminase 3 (AMPD3) gene. This is known to cause erythrocyte AMP deaminase deficiency resulting in a deficiency of a muscle isoform of the gene and is associated exercise-induced myopathy. From the dbSNP set, another arginine to cysteine substitution (R651C; rs4148876; MAF_CEU _= 0.10) which is predicted by SDM to have a large negative change in energy (-6.1), is in the antigen peptide transporter 2 (TAP2) gene. TAP2 is involved in antigen processing and the transport of antigens from the cytoplasm to a membrane-bound compartment for association with MHC class I molecules. Interestingly, a second nsSNP in this gene (T665A; rs241447; MAF_CEU _= 0.217) is also predicted to be deleterious by some of the other methods used.

**Figure 2:**
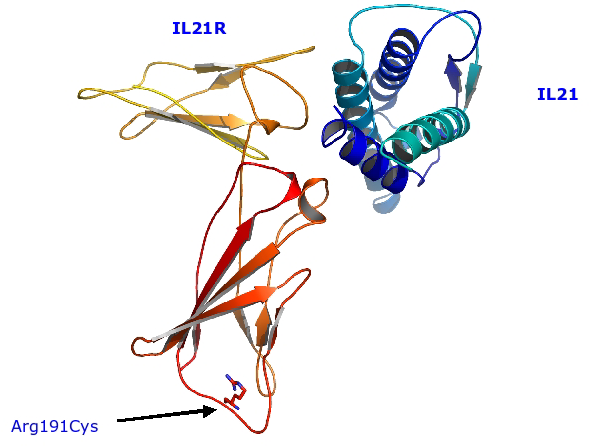
**Model of IL21R/IL21 complex**. Ribbon representation of the model of IL21R in complex with its ligand, IL21. The residue which is mutated, Arg191, is show is ball-and-stick representation.

Similarly to SDM, Imutant seems to be able to distinguish the known disease causing mutations with 12% of OMIM predicted to have a large negative (<-2) ΔΔG compared to 7% for dbSNP. One such nsSNP (Arg191Cys; rs3093370; MAFceu = 0) from the dbSNP set, is predicted to be deleterious by both SDM and Imutant as well as by SIFT, Env_score and LS-SNP occurs in the interleukin 21 receptor (IL21R). IL-21R is a novel, T-cell specific type I cytokine receptor most related to the IL-2 receptor beta chain. Figure 2 shows the model of IL-21R in complex with IL-21. The residue, Arg191 is buried and is not involved in binding of the cytokine but forms charge-charge interactions.

**Figure 3:**
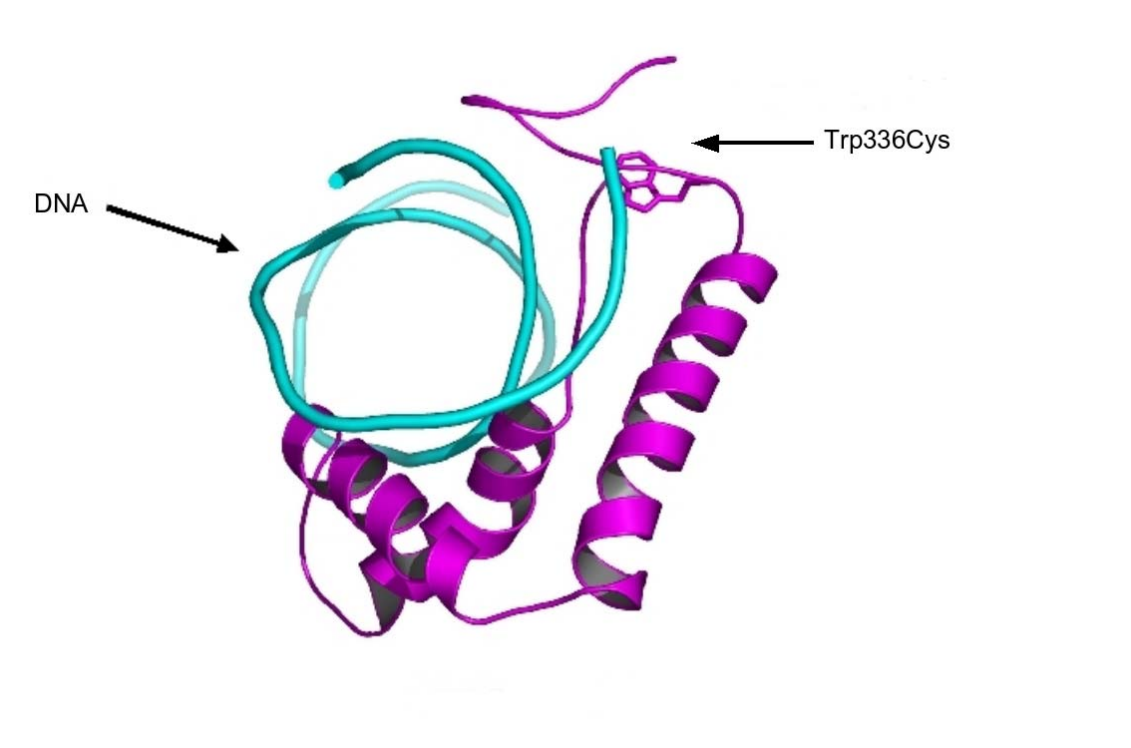
**Model of TCF7 in complex with DNA**. Ribbon representation of the model of TCF7 in complex with DNA. The residue which is mutated, Trp336, is show is ball-and-stick representation. The tryptophan residue interacts with the major groove of the DNA.

A polymorphism (Pro227Thr;MAF_CEU _= 0.12) in the TCF7 gene has previously been described to be associated with type 1 diabetes [49] and is predicted to be structurally destabilising. Another mutation (Trp336Cys; rs1135728; MAF_CEU _= unknown) in the HMG_box region of this gene is predicted to be deleterious by many methods (SIFT, env_score, SDM and Polyphen). Figure 3 shows a model of the HMG box region of TCF7 in complex with DNA. The tryptophan residue sits in the major groove of the DNA and is critical for interacting with the DNA.

### Prediction of functional residues

Functional residues can be defined as those amino acids that are directly involved in enzyme catalysis or protein-protein or protein-ligand interactions. Sometimes these interactions are well documented or can be inferred from structures of complexes of homologous structures. Indeed, these are annotated in the predictions available from LS-SNP. However, this information is often sparse and it is not straightforward to extrapolate to distantly related homologues. The models produced in this study are built as uncomplexed, monomeric structures. Some of the amino acids will be involved in interfaces if the protein exists as a dimer or higher order oligomers or they could be involved in binding to a ligand or other molecules. The analysis of sequence variations of amino acids involved in protein interfaces, inferred from complexes of close homologues, will be considered in a separate study (Bickerton, unpublished data). Here, we used Crescendo to try to predict, from sequence conservation scores, residues which may be involved in function or binding other molecules.

Crescendo was applied using the sequence alignments produced by FUGUE and the residue scores mapped onto the model of the protein. We considered only the subset of mutations that had neutral or favourable environmental substitution scores (env_score > -2) since these are less likely to result in major structural rearrangements. In general, the distribution of the mutations in the OMIM is skewed towards positive Z-scores, an indicator of functional residues, compared with the dbSNP set of mutations (data not shown). Indeed 24% of the OMIM mutations have a Z-score greater than 1.0 compared to 16% for dbSNP. Of those in the dbSNP, 1.4% (311 nsSNPs in 272 genes) were also predicted to be deleterious by LS-SNP.

One of the OMIM mutations (Asp299Gly; rs4986790; MAF_CEU _= 0.033), which is predicted by Crescendo to be functional, occurs in the Toll-like receptor 4 (TLR4) gene. TLR4 activates inflammatory gene expression through NF-kappa-B and MAPK signalling and is important in innate immune responses. This mutation is known to cause endotoxin hypo-responsiveness by affecting TLR4-mediated LPS signaling [50]. Another nsSNP (rs4740; MAF_CEU _= 0.30) from the dbSNP set is found in the Epstein-Barr virus induced gene 3 (EBI3), a protein homologous to the p40 subunit of interleukin 12 (IL-12). This is a cytokine that drives rapid clonal expansion of naive but not memory CD4 T-cells and is involved in the stimulation and maintenance of Th1 cellular immune responses, including the normal host defense against various intracellular pathogens. IL-12 also has an important role in pathological Th1 responses, such as in inflammatory bowel disease and multiple sclerosis [10,51].

### Consensus predictions

**Figure 4:**
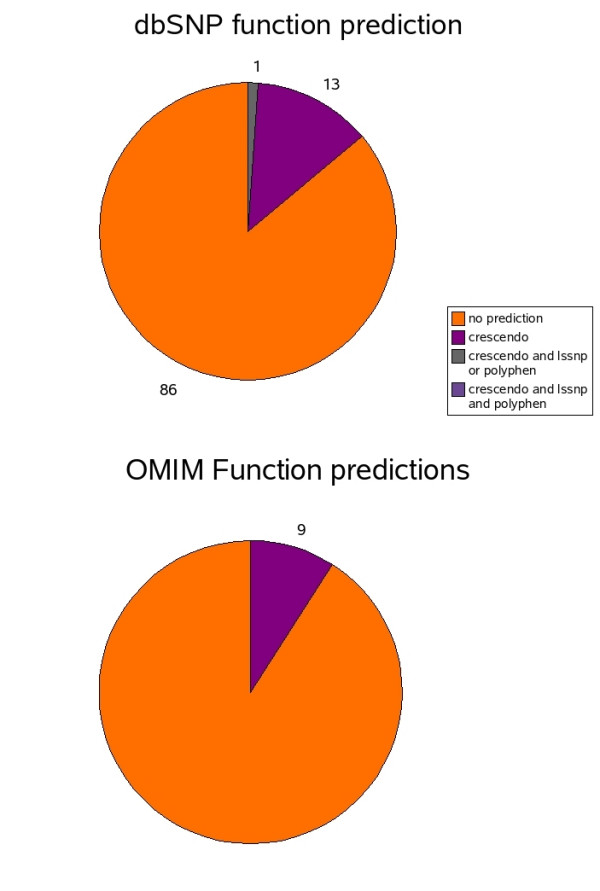
**Distribution of predicted functional residues**. Pie chart showing percentage of mutations affecting predicted functional residues for OMIM and dbSNP

**Figure 5:**
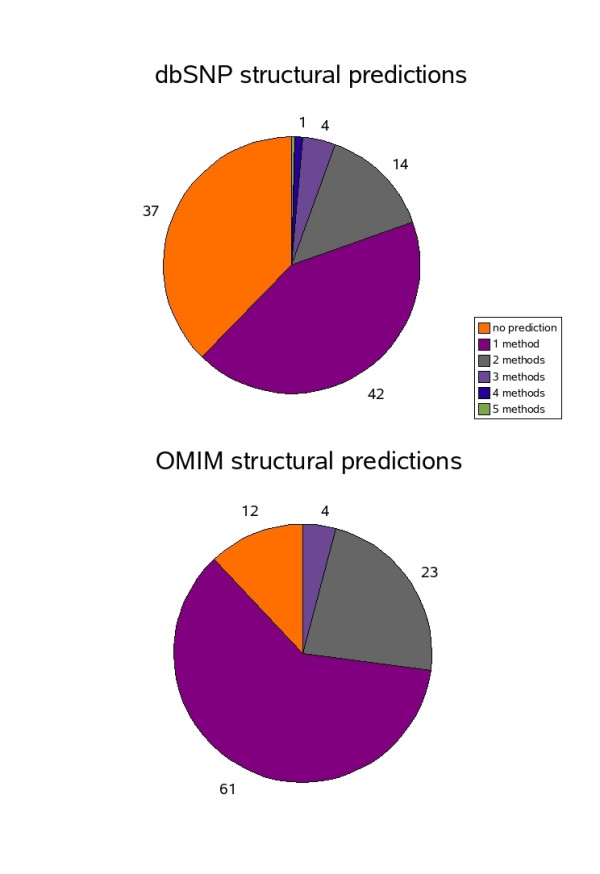
**Distribution of predicted structurally deleterious nsSNPs**. Pie chart showing percentage of structurally deleterious predictions for OMIM and dbSNP datasets.

Figures 4 and 5 shows a summary of the overlap for all of the predictions using the various methods. Almost the same percentage of OMIM mutations (9%) with structurally neutral or favourable substitutions are predicted to involve functional residues compared to those in dbSNP (13%). However, the requirement of structural information for a crescendo function prediction (resulting in predictions for only half of the mutations), means that the coverage in OMIM is small. Generally, information detailing residues involved in protein-protein interactions and ligand binding is still largely lacking. What knowledge there is available can be derived from existing experimental structures or protein-protein interaction databases. Some of this information is contained in the LS-SNP and Polyphen predictions, although collating this information is not straightforward and extrapolating to distant homologues is not always valid. This makes it is extremely difficult to produce consensus function predictions that include multiple methods. Indeed, only an additional 1% of dbSNP nsSNPs contain deleterious LS-SNP or Polyphen predictions.

Significantly more OMIM mutations are predicted to be structurally destabilising by at least two methods compared to those in dbSNP. There are 83 (3.7%) mutations in OMIM predicted to be structurally destabilising by at least three of the methods, reducing to only one mutation predicted by four of the methods. This rare mutation (Leu21Pro) occurs in the gene GTF2H5, a transcription factor involved in DNA repair, and causes trichothiodystrophy resulting in patients with brittle hair and nails, due to a reduced content of cysteine-rich matrix proteins. Considering both functional and structural predictions, an additional OMIM mutation is predicted by four of the methods. The Lys426Arg substitution in the gene ATIC causes AICA-ribosiduria resulting in disruption of purine biosynthesis by affecting the binding of a potassium ion.

### Classification by allele frequency

Each of the nsSNPs in dbSNP was correlated with the HapMap database to identify its allele frequency in the European cohort (CEU). This enables each nsSNP to be classified into one of three classes of allele frequency. Only 23% (5,770) of the nsSNPs were found in the current release of the HapMap2 database. Almost 8% (425) of these had MAFs below 0.01 and are thus considered to be rare mutations. These variations are assumed to only occur in a single individuals or specific population or in extremely rare monogenic diseases. Over 17% (960) had MAFs in the 0.01–0.05 range whereas the majority (4,385 or 76%) had MAFs > 0.05 and can be classified as common sequence variations. Over 1% of the dbSNP mutations (299) are predicted to be deleterious by four or more methods, 49 of which have been validated in HapMap [2]. Of these, 35 have a MAF_CEU _> 0.05 and are shown in table 3.

**Table 3:** Deleterious nsSNPs in dbSNP. Mutations in dbSNP with MAF > 5% predicted to be deleterious by at least four of the methods

Ensembl Gene Id	Hugoname	Gene description	dbSNP id	Mutation	MAF
ENSG00000100116	GCAT	2-amino-3-ketobutyrate coenzyme A ligase	rs710187	R39C	47
ENSG00000133048	CHI3L1	Chitinase-3 like protein 1	rs880633	R145G	47
ENSG00000148773	MKI67	Antigen KI-67	rs1063535	P2608L	47
ENSG00000168124	OR1F1	Olfactory receptor 1F1	rs1834026	F75S	44
ENSG00000147576	ADHFE1	alcoho dehydrogenase	rs1060242	C401R	42
ENSG00000122359	ANXA11	Annexin A11	rs1049550	R230C	41
ENSG00000137124	ALDH1B1	Aldehyde dehydrogenase X	rs2073478	R107L	41
ENSG00000132677	RHBG	Rhesus type B glycoprotein	rs3748569	G315R	39
ENSG00000108759	KRTHA2	Keratin, type I cuticular HA2	rs2071563	T395M	38
ENSG00000006788	MYH13	Myosin heavy chain	rs2074877	M1071V	38
ENSG00000114480	GBE1	1,4-alpha-glucan branching enzyme	rs2305246	R190G	35
ENSG00000104901	DKKL1	Dickkopf-like protein 1	rs1054770	G187S	31
ENSG00000163482	STK36	serine/threonine kinase 36 fused homolog	rs1863704	G1003D	30
ENSG00000125775	SDCBP2	Syntenin-2	rs1048621	R138C	28
ENSG00000176937	OR52R1	Olfactory receptor 52R1.	rs7941731	I128T	28
ENSG00000172071	EIF2AK3	Eukaryotic translation initiation factor 2a3	rs867529	S136C	27
ENSG00000137809	ITGA11	Integrin alpha-11	rs7168069	L524R	26
ENSG00000149305	HTR3B	5-hydroxytryptamine serotonin receptor 3B	rs1176744	Y129S	25
ENSG00000132677	RHBG	Rhesus type B glycoprotein	rs11586833	V143D	25
ENSG00000168787	OR12D2	Olfactory receptor	rs2073152	S121C	23
ENSG00000072571	HMMR	Hyaluronan mediated motility receptor	rs299284	R92C	13
ENSG00000143412	ANXA9	Annexin A9	rs267733	D159G	13
ENSG00000113108	APBB3	Amyloid beta A4 protein-binding family B3	rs250430	C240R	13
ENSG00000070371	CLTCL1	Clathrin heavy chain 2	rs807459	Y279C	12
ENSG00000070371	CLTCL1	Clathrin heavy chain 2	rs712952	R1046C	12
ENSG00000070371	CLTCL1	Clathrin heavy chain 2	rs1633399	I1394T	12
ENSG00000165799	RNASE7	Ribonuclease 7	rs1243469	H116Y	12
ENSG00000167207	CARD15	Caspase recruitment domain protein 15	rs2066844	R702W	11
ENSG00000141504	SAT2	Diamine acetyltransferase 2	rs13894	R126C	10
ENSG00000100033	PRODH	Proline oxidase	rs450046	R521Q	7.5
ENSG00000183059	unknown	Annexin A2 pseudogene 2.	rs855523	E53A	7.5
ENSG00000174942	OR5R1	Olfactory receptor 5R1	rs7111634	D121G	7.5
ENSG00000122679	RAMP3	Receptor activity-modifying protein 3	rs2074654	W56R	5.8
ENSG00000005844	ITGAL	Leukocyte adhesion glycoprotein 1	rs1064524	R214W	5.8
ENSG00000187021	PNLIPRP1	Pancreatic lipase related protein 1	rs11197744	N61D	5

A current debate in molecular genetics is how important is the contribution of rare sequence variations to an increased risk in common diseases. Many researchers consider that common variants will make the most important contribution to the inheritance of multi-factorial disorders, the Common-Disease/Common-Variant theory. A recent analysis found that there was an inverse association between the MAF of a nsSNP and deleterious functional prediction[52]. In other words, alleles that are functionally deleterious will tend to be selected against and will not exist at high frequencies. Here, we asked the question whether the nsSNPs with rare MAFs are more or less frequently predicted to be deleterious than those variations with higher allelic frequencies. If we consider the mutations with a deleterious prediction by any method (2,009 nsSNPs), over 76% had a MAF > 0.05 whilst only 9% had MAF < 0.01. These figures are virtually identical to the proportions of these classes of MAFs occurring in our dataset (76% and 8%, respectively). When we consider the 49 nsSNPs that are predicted by four or more methods, the proportion of rare alleles that are predicted to be deleterious increases to 12% whilst the common nsSNPs decreases slightly to 72%. Due to the small number of mutations involved, it is unclear whether this is a statistically significant affect.

## Discussion

For nearly 90% of the genes analysed here, we have been able to build a homology-based model of a large part of the gene which can be useful in understanding the biological effect of a mutation and its effect on function. These are based on automatically generated structure predictions. It is well documented that the accuracy of structure prediction can be improved by a careful analysis of each model[53]. This is often time consuming and so can only be applied to a small subset of these genes. Some of these genes are now being selected for further detailed modelling and structural analysis. We have also generated structure-based predictions for the effects of over 40% of the 24,000 nsSNPs observed in these genes. In addition, we have sequence-based predictions for the remaining 60% of them. We have shown that most of the prediction tools used are, in general, able identify a larger proportion of the mutations which are known to cause disease, as represented by the OMIM mutations, than compared to the largely neutral mutations in the dbSNP dataset. Using these predictions we have identified several hundred of the nsSNPs which could be involved in common disease. It is important to highlight these variants because if they are not included in the large-scale genotyping platforms, nor covered via LD with nearby SNPs that are on the genotyping platform, then it would be justified to develop special genotyping panels or individuals assays for analysis in case-control sample sets.

Currently, over 700 genes (12%) have no confident structure prediction. This lack of structural information is also a problem for regions of genes that are predicted to be disordered or have low complexity. Even where there is structural information, details of functional residues are sparse and sometimes inconsistent. There are many projects underway to improve data extraction from the plethora of biological databases or biomedical literature [54,55]. Such information will be invaluable to guide future modelling of multi-protein complexes and to improve the interpretation of mutation data. The prediction of multi-protein complexes given the structure of individual gene products will also allow us to analyse the effects of common nsSNP variants between interacting genes or multiple nsSNPs within the same gene.

We have also found evidence that rare alleles are predicted to be deleterious as often as commonly occurring alleles. These conclusions may be confounded by the fact that most of the prediction methods use the OMIM database of rare alleles as a parameterization set which may bias predictions to certain types of amino acid substitutions. The validity of many of the dimorphisms found in dbSNP is still unclear. Since only 25% of these nsSNPs have been subsequently found in sequencing projects such as HapMap, there is a potential that the majority of data in dbSNP contains erroneous, rare or population specific substitutions. Accurate estimates of the validity of the nsSNPs are needed for all available polymorphisms.

## Conclusion

It is expected that a proportion of the nsSNPs in dbSNP that are predicted to be deleterious will increase the relative risk of a developing a disease or traits but may not be sufficient to cause disease. Many other multi-genic or environmental factors will also be required before disease is evident. In the next few years, genetic studies will provide large amounts of data linking nsSNPs and other functional SNPs, and deletion-insertion polymorphisms, affecting gene expression and splicing, to the risk of developing many diseases. Detailed analysis of the effect sequence variations of the structure of the protein and its interactions will be essential for understanding the role sequence variations have in multi-factorial diseases such as cancer, type 1 diabetes and heart disease. The automated analyses and model building described here can easily be applied to new sequence variations as they become associated with susceptibility to common disorders.

## Methods

### Datasets of non-synonymous SNPs

There are two sets of mutation data available for analysis and comparison. The first set, referred to as OMIM, consists of 2,256 sequence variations found in 500 genes previously identified for Mendelian inherited disorders according to the OMIM database [14]. The second set, dbSNP, consists of 21,471 sequence variations extracted from the dbSNP database found in nearly 5,000 genes. This list of genes was derived from a list of human candidate genes related to immunity, apoptosis, or genes which are GPCRs compiled using Ensembl version 27, human genome assembly build 35 by the Juvenile Diabetes Research Foundation/Wellcome Trust Diabetes and Inflammation Laboratory at the Cambridge Institute for Medical Research. Subsequently, the OMIM genes were added to the list. In total there are 23720 unique sequence variations found in over 5500 genes.

### Homology recognition of protein sequences

Homology recognition for all of the genes was performed using the program FUGUE[38]. FUGUE is a program for recognizing distant homologues by sequence-structure comparison. Given a sequence or a sequence alignment, it scans a database of structural profiles derived from the HOMSTRAD database and calculates a compatibility score and produces a list of potential homologues and alignments. FUGUE uses environment-specific substitution tables and structure-dependent gap penalties. For every gene sequence, homologous sequences were collected using PSI-BLAST. The multiple sequence alignment was used as input to FUGUE. Only potential homologues with a score greater than 7 were considered. For potential hits with overlapping sequence alignments, the lowest scoring were removed.

### Structure prediction using comparative modelling

Once an evolutionary relationship has been established to a HOMSTRAD family, and hence to a protein of known 3D structure, a model of the structure of the protein can be built using comparative modelling techniques[52,56]. The sequence-structure alignment given by FUGUE was used to build a model with the program Modeller[42]. Due to the large number of alignments, no re-alignment of the FUGUE alignment was attempted.

### Environmental substitution scores

Residue environments are defined, as described by Topham [57], in terms of mainchain conformation, relative sidechain solvent accessibility and sidechain hydrogen bonding. Four mainchain conformations (α-helix, b-strand, +ve phi and coil) are defined along with two types of solvent accessibility (<40%; over 40%). There are also three types of independent hydrogen bonding possible (sidechain to sidechain; sidechain to mainchain amide; sidechain to mainchain carboxyl). This gives a total of 64 (4 × 2 × 2 × 2 × 2) residue environments. These tables describe the probabilities of substituting a residue in each structural environment by each of the 21 residue types, distinguishing cystine and cysteine.

### Prediction of functional residues by crescendo

Crescendo [30] identifies amino acids substitutions which are likely to be involved in protein function or protein interactions. It compares the observed sequence conservation for each amino acid position in the homologous sequences of a protein with the conservation pattern predicted on the basis of local environment substitution tables. For those positions where the environment substitution tables make poor predictions of the overall amino acid substitution pattern, it can be assumed that these regions must be restrained by functional requirements of the protein. The Crescendo score quantifies the degree of observed sequence conservation at an amino acid position compared to the conservation expected based upon the environment-dependent substitution tables.

### Prediction of free energy change by SDM

Protein stability is dependent on the difference in free energy (ΔG^U-F^) between the folded (F) and unfolded (U) states (ΔG^U-F ^= G^U ^- G^f^). The mutation of a residue of type j in the wild-type protein to residue of type k may be coupled to the reversible folding-unfolding process by means of the thermodynamic cycle. The difference in free energy of unfolding, ΔΔG, of the wild type and mutant, is therefore related to the respective free energy changes associated with the transformation of j-> k in the unfolded and folded states through the thermodynamic cycle. SDM calculates stability score differences (Δs), derived from environmental substitution tables[36,37], for mutations in the folded and unfolded structural environments. Analogous to the thermodynamic cycle, these scores can be then be used to represent the component processes of the folding-unfolding cycle to predict a ΔΔs for a mutation[16].

## Abbreviations

nsSNPs non-synonymous single nucleic polymorphisms

MAF_CEU _Minor allele frequency of an allele in the Central European Cohort.

## Authors' contributions

DFB carried out the studies and analysis. DFB and TLB conceived of the study and participated in its design and coordination. DFB, LJS, JAT and TLB helped to draft the manuscript. All authors read and approved the final manuscript.
